# Evaluation of Ambient Sensor Systems for the Early Detection of Heart Failure Decompensation in Older Patients Living at Home Alone: Protocol for a Prospective Cohort Study

**DOI:** 10.2196/55953

**Published:** 2024-05-31

**Authors:** Benjamin Vögeli, Nisha Arenja, Narayan Schütz, Tobias Nef, Philipp Buluschek, Hugo Saner

**Affiliations:** 1 Department of Cardiology Solothurner Spitäler AG, Kantonsspital Olten Olten Switzerland; 2 Faculty of Medicine University of Basel Basel Switzerland; 3 Stanford School of Medicine Stanford University Stanford, CA United States; 4 ARTORG Center for Biomedical Engineering Research University of Bern Bern Switzerland; 5 DomoHealth SA Lausanne Switzerland

**Keywords:** heart failure, home telemonitoring, digital health, biomarker, ambient sensor system

## Abstract

**Background:**

The results of telemedicine intervention studies in patients with heart failure (HF) to reduce rehospitalization rate and mortality by early detection of HF decompensation are encouraging. However, the benefits are lower than expected. A possible reason for this could be the fact that vital signs, including blood pressure, heart rate, heart rhythm, and weight changes, may not be ideal indicators of the early stages of HF decompensation but are more sensitive for acute events triggered by ischemic episodes or rhythm disturbances. Preliminary results indicate a potential role of ambient sensor–derived digital biomarkers in this setting.

**Objective:**

The aim of this study is to identify changes in ambient sensor system–derived digital biomarkers with a high potential for early detection of HF decompensation.

**Methods:**

This is a prospective interventional cohort study. A total of 24 consecutive patients with HF aged 70 years and older, living alone, and hospitalized for HF decompensation will be included. Physical activity in the apartment and toilet visits are quantified using a commercially available, passive, infrared motion sensing system (DomoHealth SA). Heart rate, respiration rate, and toss-and-turns in bed are recorded by using a commercially available Emfit QS device (Emfit Ltd), which is a contact-free piezoelectric sensor placed under the participant’s mattress. Sensor data are visualized on a dedicated dashboard for easy monitoring by health professionals. Digital biomarkers are evaluated for predefined signs of HF decompensation, including particularly decreased physical activity; time spent in bed; increasing numbers of toilet visits at night; and increasing heart rate, respiration rate, and motion in bed at night. When predefined changes in digital biomarkers occur, patients will be called in for clinical evaluation, and N-terminal pro b-type natriuretic peptide measurement (an increase of >30% considered as significant) will be performed. The sensitivity and specificity of the different biomarkers and their combinations for the detection of HF decompensation will be calculated.

**Results:**

The study is in the data collection phase. Study recruitment started in February 2024. Data analysis is scheduled to start after all data are collected. As of manuscript submission, 5 patients have been recruited. Results are expected to be published by the end of 2025.

**Conclusions:**

The results of this study will add to the current knowledge about opportunities for telemedicine to monitor older patients with HF living at home alone by evaluating the potential of ambient sensor systems for this purpose. Timely recognition of HF decompensation could enable proactive management, potentially reducing health care costs associated with preventable emergency presentations or hospitalizations.

**Trial Registration:**

ClinicalTrials.gov NCT06126848; https://clinicaltrials.gov/study/NCT06126848

**International Registered Report Identifier (IRRID):**

PRR1-10.2196/55953

## Introduction

The results of telemedicine intervention studies in patients with heart failure (HF) to reduce rehospitalization rate and mortality by early recognition of HF decompensation are encouraging [1-7]. However, benefits have been found to be lower than expected. Therefore, according to the European Cardiology Guidelines, home telemonitoring (HTM) of patients with HF is a class IIb indication, while in the American Guidelines, there is no recommendation for HTM [8,9]. One reason that previous studies with HTM have failed to show greater benefits may be that vital signs, including blood pressure, heart rate, heart rhythm by rhythm strips, and weight changes, may not be ideal indicators of the early stages of HF decompensation but may be more sensitive for acute events such as ischemic episodes or rhythm disturbances.

Recently, early detection of impending HF hospitalization through continuous wearable monitoring analytics has been described [10]. However, wearables are difficult to use in some patient populations, as good compliance is essential. This can be particularly challenging for older adults or individuals with limited digital literacy or cognitive impairment, as devices need to be recharged daily and have rather small screens and complex user interfaces [11].

Ambient sensor systems (ASSs) are increasingly used to monitor older people in their apartments not only for safety but more recently for monitoring physiological factors such as physical activity and detection of health problems such as frailty, cognitive impairment, depression, social isolation, sleep, pulmonary emboli, heart rhythm disturbances, and COVID-19–related health deteriorations [12-17]. Preliminary data indicate that an ASS in the home of older patients with HF living alone has the potential to indicate early stages of decompensated acute HF [18].

The aim of this prospective interventional cohort study is to evaluate the sensitivity and specificity of a set of ASS-derived digital biomarkers with the highest potential for early detection of HF decompensation, allowing timely intervention to prevent serious health deterioration and rehospitalization.

## Methods

### Study Design and Participants

This is a prospective, interventional cohort study. In total, 24 consecutive patients living alone at their homes and with hospitalization for HF decompensation at the 2 Solothurner Spitäler AG secondary care hospitals in Olten and Solothurn (Switzerland) will be included.

The study inclusion criteria are current hospitalization for HF decompensation*,* aged 70 years and older, left ventricular ejection fraction (LVEF) <50%, treatment with diuretics, New York Heart Association classification II or III, living alone, willingness to participate with informed consent, and agreement for follow-up appointments in the hospital at 3 and 6 months.

Exclusion criteria include major depression (Patient Health Questionnaire-9 score>9) or being on hemodialysis. In addition, patients with a left ventricular assist device or those who had undergone coronary revascularization or cardiac resynchronization therapy implantation within 28 days before the index event of HF decompensation or have been scheduled for such interventions are excluded from the study.

### Ethical Considerations

The study will be conducted based on the principles expressed in the Declaration of Helsinki, the International Council for Harmonisation of Technical Requirements for Pharmaceuticals for Human Use—Good Clinical Practice, and the Human Research Act after protocol approval by the regional ethics committee (Ethikkommission Nordwestschweiz; Swissethics BASEC ID 2023-02138). Patients will provide written informed consent. Participants can withdraw from the study at any time during the study process. Data that were collected until withdrawal will still be analyzed. Data will be collected by specially trained research staff and entered into a password-protected data environment. Each patient will be attributed a study-specific patient identifier (PID). For statistical analysis, these data sets will be merged using the PID as an identifier. At the end of the data acquisition, including follow-up, patient data will be coded using the PID, and the database will be locked. Coding using the PID will be done at the earliest time point after the completion of follow-up data collection. Data generation, transmission, storage, and analysis of health-related personal data within this study will follow the current Swiss legal requirements for data protection and will be performed according to the Ordinance HRO Art 5. Health-related personal data captured during this study is strictly confidential, and disclosure to third parties is prohibited. Participants will be compensated for costs resulting from travel expenses due to follow-up visits in the hospital. For study participation, no costs incur for participants or health insurance companies.

### Baseline Assessment

The following baseline parameters will be evaluated at hospital entry: age, gender, BMI, medical history, diagnosis (ischemic or nonischemic origin of cardiomyopathy), current medication, as well as N-terminal pro b-type natriuretic peptide (NT-proBNP) and 2D echocardiography after decongestion. LVEF will be calculated according to current guidelines [19]. A 6-minute walking test and isometric hand strength test (handgrip test) will be performed before hospital discharge.

### Ambient Sensor System

**Figure 1:**
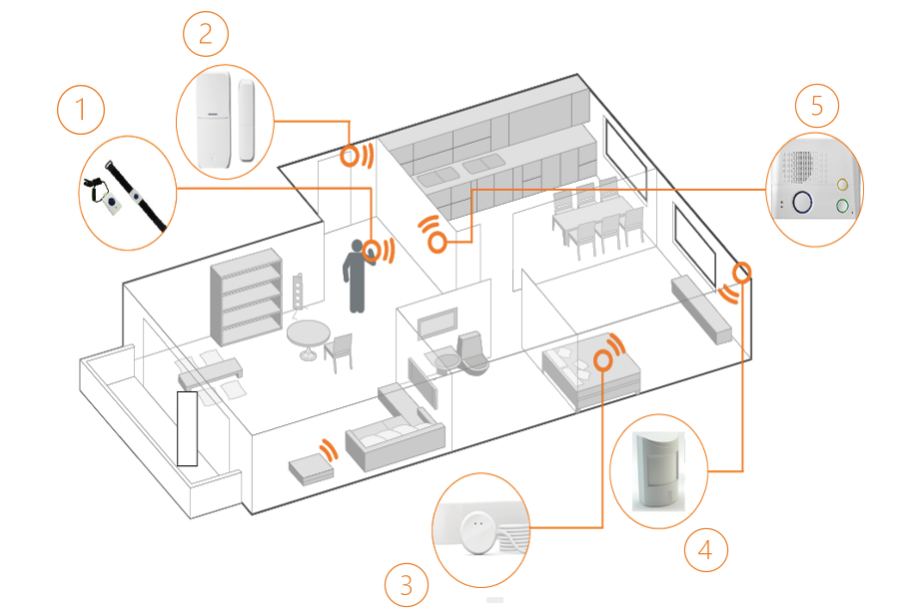
Graphical representation of the ambient sensor system used in this study: (1) alarm button, (2) door sensor, (3) Emfit QS bed sensor, (4) passive infrared motion sensor in each room (including bathroom), and (5) interphone.

Physical activity in the apartment and bathroom visits are quantified using a commercially available passive infrared (PIR) motion sensing system and visualized on a dashboard (DomoHealth SA). The system includes PIR motion sensor units for each room that is used in the home and 2 magnetic door sensors for entry-door and fridge-door openings that communicate wirelessly with a base unit (Figure 1). Motion activation is measured with a resolution of 1 second. The absence of motion is notified when no motion is recorded for 30 seconds. All sensors communicate with the base unit through an encrypted radiofrequency protocol. The base unit collects the data and sends them to the cloud in real time using the Global System for Mobile Communications network. The participants’ kitchen, living room, entrance, bedroom, and bathroom will be equipped with 1 PIR sensor based on the room size of the patients’ apartment. Motion signals are collected room by room and combined as the total activity in the apartment. Normalized daily PIR sensor activity refers to the time the PIR sensors are registering activity at home, normalized by the total time spent at home for a given day. It is thus corrected for the influence of time spent out of home. A bathroom visit is identified as such if, in a 30-minute window, at least 1 sensor firing in the bathroom is recorded. Nighttime is defined as the period from 9:30 PM to 8 AM (local time) based on the daily activity habits of the patients. Heart rate, heart rate variability, respiration rate, and presence are recorded in bed with the commercially available Emfit QS device (Emfit Ltd), a contact-free piezoelectric sensor placed under participants’ mattresses. These sensors use thin quasi-piezoelectric films that measure even light pressure differences produced by the beating heart. The Emfit QS sensor will be fixed under the participants’ mattress, in proximity to the chest. The device requires no further manual intervention. Data will be transmitted to the cloud in real time through local Wi-Fi and, subsequently, the Global System for Mobile Communications network. The device extracts a variety of vital signs, including heart rate, respiration rate, heart rate variability, movements in bed, sleep duration, and sleep onset delay. Recent results suggest that this sensor can accurately measure heart rate and respiration rate [20]. The reliability and potential of the use of ASS-derived digital biomarkers as indicators for health conditions and diseases have been shown in several publications by members of our research group [12-18,21-25].

Sensor data are then visualized on a dedicated dashboard (entry door openings, activity in different rooms, fridge openings, bathroom visits, and bed presence), and the DomoCare (DomoHealth SA) app (heart rate, respiration rate, sleep quality, and preventive warnings) can be used by the investigator team to easily monitor the health and behavior of a system user. Sensor-derived digital biomarkers are summarized in Table 1.

**Table 1:** Ambient sensor–derived digital biomarkers.

Biomarker		Sensor
**Disease-specific digital biomarker**		
	Number of toilet visits at night	PIR^a^ motion sensor+Emfit QS bed sensor
	Average heart rate in bed	Emfit QS bed sensor
	Average respiration rate in bed	Emfit QS bed sensor
**Unspecific digital biomarker**		
	Daily activity	PIR motion sensor
	Number of toss and turns in bed	Emfit QS bed sensor
	Time spent in bed	Emfit QS bed sensor
	Number of refrigerator openings	Contact sensor

^a^PIR: passive infrared.

### Outcome Assessment

#### Ambient Sensor–Derived Digital Biomarkers

Based on previous experience from the use of the system in older patients with HF, the changes (based on a 1-week moving average) of the measured parameters (presented in Table 2) over 3 weeks will be used as potential indicators of HF deterioration.

**Table 2:** Potential indicators of heart failure deterioration.

Indicators	Values
PIR^a^ motion sensor–derived daily activity	–1000 units or –30%
Time spent in bed	+20%
PIR motion sensor–derived number of toilet visits at night	+30%
Emfit QS bed sensor–derived number toss and turns at night	+30%
Emfit QS bed sensor–derived average heart rate (night)	+3 bpm with β-blocker or +6 bpm without β-blocker
Emfit QS bed sensor–derived average respiration rate increase (night)	≥5 per minute

^a^PIR: passive infrared.

#### Indicators for Significant Health Deterioration

If the dashboard reports relevant changes, as described before, in at least 1 specific and 2 unspecific digital measures or 2 specific measures, the patient will be contacted and called in for a visit by the investigator team to check for changes from the last visit. We will use an NT-proBNP increase of >30% compared to the measurement after recompensation at hospital discharge as confirmation for health deterioration. This will be called successful event detection for the ASS or the respective digital biomarker [26].

#### Primary Outcome

The primary outcome of the study will be the sensitivity and specificity of different ambient sensor–derived digital biomarkers to recognize HF decompensation (defined as an NT-proBNP increase of ≥30%).

#### Secondary Outcome

The secondary outcomes include outcome data such as the total number of rehospitalizations for HF decompensation, the total number of HF decompensations requiring medical intervention, and cardiovascular and all-cause death over the follow-up period (Table 3). We will also evaluate the quality of life measured through the Kansas City Cardiomyopathy Questionnaire, as well as functional status (6-minute walking test and handgrip strength test) and changes in NT-proBNP values at follow-up visits after 3 and 6 months.

**Table 3:** Timeline of follow-up data.

Outcome	Baseline	3 months	6 months
LVEF^a^	✓		
NT-proBNP^b^	✓	✓	✓
6MWT^c^	✓	✓	✓
Handgrip test	✓	✓	✓
KCCQ^d^	✓	✓	✓

^a^LVEF: left ventricular ejection fraction.

^b^NT-proBNP: N-terminal pro b-type natriuretic peptide.

^c^6MWT: 6-minute walking test.

^d^KCCQ: Kansas City Cardiomyopathy Questionnaire.

### Statistical Analysis

There are no previous data to be used for a regular power calculation. The selected number of 24 study patients is based on experience from a previous study including a similar patient population with hospitalization for HF decompensation. This study showed that approximately 30% of HF decompensation with rehospitalization without HTM intervention is to be expected within 6 months after hospital discharge in this patient population [27].

We will count the true-positive and false-positive indicators for a significant event (>30% increase of NT-proBNP) for the calculation of the sensitivity and specificity of the respective digital biomarkers.

**Figure 2:**
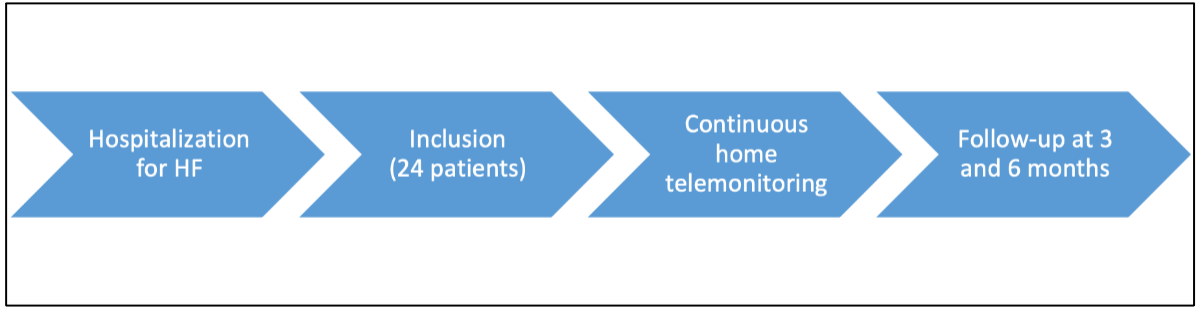
Timeline of the study procedure. HF: heart failure.

The completion of the study will be achieved if the number of 24 patients has been reached or if an interim analysis of the first 10 patients shows sensitivity or specificity of the digital biomarkers for impending HF decompensation of ≤60% at the 3-month evaluation. A timeline of the study procedure is provided in Figure 2.

## Results

The study is in the data collection phase. Study recruitment started in February 2024. Data analysis is scheduled to start after all the data are collected. As of manuscript submission, 5 patients have been recruited. Results are expected to be published by the end of 2025.

## Discussion

### What Does the Study Offer?

The use of telemedicine with home monitoring of patients with HF has been well studied under various circumstances [1-7,28-30]. In Germany, telemedical surveillance of patients with HF is even covered by health insurance. Especially in patients with chronic disease, where a regular follow-up is beneficial, telemedicine seems to be a favorable way of patient management due to the possibility to detect health deterioration in the early stages. In terms of chronic HF, the worsening of HF symptoms or ultimately HF decompensation could be intercepted by regular telemedical follow-up visits with the possibility of making early adjustments to the therapy and through that potentially preventing hospital admissions. Remote patient management includes observation and determination of disease seriousness by patient feedback and, for example, the collection of data on vital parameters (eg, heart rate, blood pressure, blood oxygen saturation, and bodyweight) and activity status. However, the commonly used approach to telemedicine in patients with HF includes self-measurements of blood pressure, heart rate, single-lead electrocardiogram, and body weight, which carries a significant risk of noncompliance.

This is to our knowledge the first prospective study investigating ASS-derived digital biomarkers in patients with HF. Contact-free ASS has the advantage that they can be unrestrictedly used even in a patient population with moderate to severe cognitive impairment as they bypass user-dependent sources of error [21]. Clinical signs of HF decompensation include shortness of breath, rhythm disturbances, fatigue and weakness, reduced ability to exercise, edema, and nycturia. Most of these can be detected by ASS, and associated signals or a combination of them may be used as digital biomarkers for HF decompensation.

Preliminary data have been promising in the detection of significant health deterioration using unobtrusive ASS. This study offers insights into the effectiveness of the system to detect HF decompensation in the context of an older patient population with HF who are particularly at risk for frequent HF decompensations and rehospitalizations.

### Limitations

A prospective cohort is most accurate for discovering predictive biomarkers. However, this study has several limitations. First, these markers or combinations must be validated internally and externally. Second, the 2-center nature of this study may imply selection bias. A third limitation of the study, concerning primarily the use of ASS, is that its operating area is limited to patients who live alone. Patients who live in multiperson households may display different behaviors within the residential environment. This complicates the generalizability of the investigated digital biomarker thresholds to other HF populations. The small sample size is based on the “proof-of-concept” design of this study.

### Conclusions

This study aims to provide a new set of ASS-derived digital biomarkers for early recognition of HF decompensation in an older population with HF with reduced LVEF. Accurate prediction of impending HF decompensation will allow for proactive management of patients with HF, with the possibility to reduce HF-related worsening of health conditions and thus reduce health care costs associated with preventable emergency department presentations or even hospitalizations. If the “proof-of-concept” is positive, the concept of using ASS-derived biomarkers for early detection of signs of HF decompensation should be further investigated in larger prospective studies.

## Abbreviations

ASS: ambient sensor system
HF: heart failure
HTM: home telemonitoring
LVEF: left ventricular ejection fraction
NT-proBNP: N-terminal pro b-type natriuretic peptide
PID: patient identifier
PIR: passive infrared

## Data Availability

The data sets generated and analyzed during this study are available from the corresponding author on reasonable request.
